# There are alternatives. Models for sustainable employment structures in the German system of higher education

**DOI:** 10.3389/frma.2024.1301354

**Published:** 2024-03-01

**Authors:** Mathias Kuhnt, Peter Müßig, Tilman Reitz

**Affiliations:** ^1^Laboratory for Research in Organization and Differentiation, Faculty of Arts, Humanities and Science, Dresden University of Technology, Dresden, Germany; ^2^Research Infrastructure and Methods, German Centre for Higher Education Research and Science Studies (DZHW), Hannover, Germany; ^3^Institute of Sociology, Faculty of Arts and Humanities, Friedrich-Schiller-University Jena, Jena, Germany

**Keywords:** German universities, employment statistics, employment structure, tenure-track, lecturer

## Abstract

The German system of higher education and public research is characterized by a high rate of temporary contracts with short contract durations and a nearly complete absence of structured career options. About 82% of employees not holding a full professorship have fixed-term contracts, with an average contract period of 20 months. This is facilitated by a special fixed-term employment law (Wissenschaftszeitvertragsgesetz) which universities and other public research institutions have stretched to its limits. Against the background of a survey which we conducted in 2021 and whose results once more demonstrate the shortcomings of this system, we discuss alternative options in the form of model calculations. We propose a reform of employment structures and career paths that could improve not only personal working conditions but also ensure the quality of research and teaching in German academia. By quantitative comparison with the current employment situation, our model calculation demonstrates that plannable career decisions can be enabled at an early stage without changing budgets or teaching duties. We also show that the counter argument of a “congestion” of positions is not substantiated, and that young scholars will still have the opportunity to start a career in the reformed system, while the total number of employees can be kept nearly constant.

## 1 Introduction

It is widely acknowledged that the German higher education system with its overabundance of fixed-term contracts and a lack of clear and transparent career paths is in need of reform. Only 13% of academic staff members of German universities hold a professorship, which is the only position formally equipped with a permanent contract. The remaining academic personnel are often referred to as “Wissenschaftlicher Nachwuchs” (academic offspring) and therefore not yet regarded as fully-fledged academics despite their indispensable role in teaching and research. In almost all cases, the title of not being a fully-fledged researcher serves as a reason to award only fixed-term contracts. 82% of all non-professorial staff work under these conditions (ICEland 60402, own calculations).^1^ This academic phase of early adolescence can extend well to a person's mid-40s, taking into account that the average age of the first professorial appointment is 42.5 (BuWiN – Konsortium Bundesbericht Wissenschaftlicher Nachwuchs, 2021, p. 91, estimated for W2 and W3 positions).

Discussions about limiting the rate of fixed-term contracts in German academia reach back decades now. Yet both federal and state legislators as well as universities struggle to implement substantial reforms. Ministries on the one hand acknowledge problems but, on the other, share some arguments that have hindered reforms so far. Some of these arguments have lost their appeal, such as the need for a broad throughput of fresh staff to constantly generate new ideas. Others have not.

Even if it is no longer in vogue to speak of “congestions” of the academic system, the argument—under the new guise of ensuring opportunities for new generations—is still pervasive in discussions. Too many permanent contracts are deemed to make it impossible for young academics to find any employment in academia after completing their doctorate. Interestingly, doctoral students themselves do not share these concerns and rather favor the creation of permanent positions for those holding a PhD (Kuhnt et al., 2022, p. 92). A similar argument against creating more reliable perspectives and job security is that such a system would be too expensive and consequently exclude too many people from working in academia.

These arguments against creating a more sustainable employment structure at German universities have never been proven. Much less have they been weighted against the downsides of such a policy: encroachment on employees' rights, brain drain, inefficiencies, quality problems, incentives for fraud or abuse of power, to just name a few. In this article, we demonstrate that sustainable job structures are possible without preventing new generations from finding jobs in academia and without altering universities' budgets. Moreover, we show that new employment structures would not only ensure a healthy balance between experienced researchers and doctoral students, but also significantly increase teaching outputs, so that studying conditions could be improved.

To carry out this demonstration, the following section first provides a brief introduction to the German academic system. Then, in section three, a statistical model of the current or *status quo* employment structure is constructed, and several indicators are introduced for evaluating the status quo as well as alternative models. These alternative models are presented and evaluated in section four, followed by a conclusion in section five.

## 2 German universities and their employment structure

German universities are characterized by an atomized structure of individual professors with a large degree of independence, who usually head rather small units of academic staff. This staff depends on professors in terms of contract renewals, of the supervision and evaluation of doctoral or habilitation theses. In addition to this basic structure, for about two decades so-called “junior” professorships which should lead to full professorships have been a central element in debates about improving career opportunities for academic staff. Despite some efforts to establish “junior” professorships—with or without tenure-track options—especially by the federal government, these positions continue to be quantitatively insignificant. In 2021, junior professorships amounted to a share of only 0.8% of academic employees (without full professors) and of 3.4% of full professors (ICEland 60302, own calculations, Table 1). Despite a lack of statistical data, it is safe to say that of this marginal number, only a fraction benefits from tenure track regulations (DGJ – Deutsche Gesellschaft Juniorprofessur, 2017).

**Table 1:** Key figures of German universities.

	**Value**	**Source**
**General**		
**Transition ages**		
Conclusion of studies	26.6	ICEland 34501
Conclusion of doctorate	32.2	ICEland 34501
Assumption of professorship	42.5	Own calculations, BuWiN – Konsortium Bundesbericht Wissenschaftlicher Nachwuchs, 2021, p. 91
Retirement	67	–
**Total numbers**		
Professors	25,645	ICEland 60402
Acedemic staff (budgetary, permanent)	30,470	ICEland 60402
Acedemic staff (budgetary, fixed-term)	83,755	ICEland 60402
Acedemic staff (project-funding, permanent)	1,365	ICEland 60402
Acedemic staff (project-funding, fixed-term)	75,520	ICEland 60402
Teaching staff (LfbA, permanent)	4,555	ICEland 60402
Teaching staff (LfbA, fixed-term)	1,935	ICEland 60402
**Average paid weekly hours**		
Postdoc (permanent)	0.93	*N* = 367, Kuhnt et al., 2022
Postdoc (fixed-term)	0.89	*N* = 1,307, Kuhnt et al., 2022
Teaching staff (LfbA, permanent)	0.85	*N* = 80, Kuhnt et al., 2022
Teaching staff (LfbA, fixed-term)	0.75	*N* = 28, Kuhnt et al., 2022
Predoc (fixed-term)	0.78	*N* =1,478, Kuhnt et al., 2022
**Others**		
Share postdocs	0.36	Own calculations, ICEland 60502
Share buget funding fixed-term contracts	0.52	Own calculations, ICEland 60402
**Salaries**		
Professors	114,300 €	Own calculations based on TVL E13 (2021), www.oeffentlicher-dienst.info, https://www.steuertipps.de/service/rechner/brutto/netto/gehaltsrechner/arbeitgeber/, cf. Supplementary Table 1
Postdoc (permanent)	88,764 €	
Postdoc (fixed-term)	80,684 €	
Lecturer	87,355 €	
Lecturer (10 years)	72,252 €	
Predoc	68,610 €	
**Social sciences**		
**Total numbers**		
Professors	6,150	ICEland 60402
Acedemic staff (budgetary, permanent)	1,785	ICEland 60402
Acedemic staff (budgetary, fixed-term)	14,770	ICEland 60402
Acedemic staff (project-funding, permanent)	130	ICEland 60402
Acedemic staff (project-funding, fixed-term)	8,075	ICEland 60402
Teaching staff (LfbA, permanent)	685	ICEland 60402
Teaching staff (LfbA, fixed-term)	650	ICEland 60402
**Average paid weekly hours**		
Postdoc (permanent)	0.87	*N* = 36 (social s.), Kuhnt et al., 2022
Postdoc (fixed-term)	0.88	*N* = 209 (social s.), Kuhnt et al., 2022
Teaching staff (LfbA, permanent)	0.85	*N* = 80 (all), Kuhnt et al., 2022
Teaching staff (LfbA, fixed-term)	0.75	*N* = 28 (all), Kuhnt et al., 2022
Predoc (fixed-term)	0.73	*N* = 328 (social s.), Kuhnt et al., 2022
**Others**		
Share postdocs	0.24	Own calculations, ICEland 60502
Share buget funding fixed-term contracts	0.65	Own calculations, ICEland 60402
**Engineering sciences**		
**Total numbers**		
Professors	3,810	ICEland 60402
Acedemic staff (budgetary, permanent)	2,505	ICEland 60402
Acedemic staff (budgetary, fixed-term)	10,595	ICEland 60402
Acedemic staff (project-funding, permanent)	605	ICEland 60402
Acedemic staff (project-funding, fixed-term)	21,170	ICEland 60402
Teaching staff (LfbA, permanent)	175	ICEland 60402
Teaching staff (LfbA, fixed-term)	50	ICEland 60402
**Average paid weekly hours**		
Postdoc (permanent)	0.95	*N* = 19 (engin.), Kuhnt et al., 2022
Postdoc (fixed-term)	0.95	*N* = 42 (engin.), Kuhnt et al., 2022
Teaching staff (LfbA, permanent)	0.85	*N* = 80 (all), Kuhnt et al., 2022
Teaching staff (LfbA, fixed-term)	0.75	*N* = 28 (all), Kuhnt et al., 2022
Predoc (fixed-term)	0.93	*N* = 114 (engin.), Kuhnt et al., 2022
**Others**		
Share postdocs	0.18	Own calculations, ICEland 60502
Share buget funding fixed-term contracts	0.33	Own calculations, ICEland 60402
**Natural sciences**		
**Total numbers**		
Professors	5,690	ICEland 60402
Acedemic staff (budgetary, permanent)	4,300	ICEland 60402
Acedemic staff (budgetary, fixed-term)	13,160	ICEland 60402
Acedemic staff (project-funding, permanent)	1,010	ICEland 60402
Acedemic staff (project-funding, fixed-term)	19,700	ICEland 60402
Teaching staff (LfbA, permanent)	420	ICEland 60402
Teaching staff (LfbA, fixed-term)	235	ICEland 60402
**Average paid weekly hours**		
Postdoc (permanent)	0.95	*N* = 19 (engin.), Kuhnt et al., 2022
Postdoc (fixed-term)	0.9	*N* = 42 (engin.), Kuhnt et al., 2022
Teaching staff (LfbA, permanent)	0.85	*N* = 80 (all), Kuhnt et al., 2022
Teaching staff (LfbA, fixed-term)	0.75	*N* = 28 (all), Kuhnt et al., 2022
Predoc (fixed-term)	0.7	*N* = 114 (engin.), Kuhnt et al., 2022
**Others**		
Share postdocs	0.34	Own calculations, ICEland 60502
Share buget funding fixed-term contracts	0.4	Own calculations, ICEland 60402

For the vast majority of academic staff, the path to a full professorship or any other permanent employment is mainly left to chance, and selection is often rather based on perseverance than performance. The current fixed-term contract law for academia (Wissenschaftszeitvertragsgesetz, WissZeitVG) in general allows for a temporary employment of 6 years after graduation in order to acquire a doctoral degree or any^2^ other forms of qualification, and another 6 years of temporary employment after the completion of a doctorate for further qualification, usually intended to lead to a habilitation^3^ or equivalent merits. After these 12 years, academics are on average 39 years old, if we add 12 years to the average age at which students complete a master's degree or equivalent. Considering that a full professorship is reached at an average age of 42.5 years (BuWiN – Konsortium Bundesbericht Wissenschaftlicher Nachwuchs, 2021, p. 91, estimated for W2 and W3 positions), this leaves a gap which is usually filled with temporary project funded positions. The limits restricting the total time of temporary employment do not apply for these positions. In general, temporary employment tends to be short term, with an average contract duration of 20 months (Sommer et al., 2022, p. XI). These time spans are usually too short to meet official qualification goals. Our evaluation of practices under the WissZeitVG (Kuhnt et al., 2022, p. 38) showed that postdocs in particular often do not seriously aspire to a habilitation or equivalent, although that is the official reason for their fixed-term employment.

The proportions alone −87% of all academic staff compared to 13% of professors—indicate that non-professorial staff occupy a rather hybrid role (Bloch et al., 2023) and contribute to fundamental functions of the university, involving not only research and teaching but also so-called self-administration (universitäre Selbstverwaltung) and other organizational tasks.

For what we address here as academic staff, official statistics (ICEland 60302) show a large variety of formal positions apart from or below a professorship. The most common position, at 80%, is the designation as research associate (wissenschaftliche Mitarbeiterin/wissenschaftlicher Mitarbeiter, WMA). All other formal positions like “research assistant” (wissenschaftliche Assistentin/wissenschaftlicher Assistent) are largely due to historical developments and special policies in some of the German states. The proportion of fixed-term contracts (85%, Iceland 60402) within the group of these other designations does not differ much from that of WMAs (83%), although they presumably have longer work experience. We will therefore consider them as one group.

Recent public debates on the German higher education system also refer to the different career phases (R1–R4) for academic staff as proposed in the European framework for research careers, with R1 comprising predocs, R2 (very) early postdocs, R3 later, more independent postdocs and R4 full professorships. These phases so far find little reflection in current employment situations in Germany. In modeling academic employment structures, however, a distinction should at least be made between pre- and postdocs, even though their working contracts do not differ in kind and it usually remains undecided for postdocs whether they are still working to qualify for permanent employment at a university or will have to look for work in other sectors of the economy. Unfortunately, the lack of clear demarcations in current employment situations finds continuance in most statistical data, which makes it difficult, in general and for the status quo presented here, to determine how many employees can continue with a university career after having obtained a doctoral degree and how many people compete for a full professorship at some point much later in their career.

Furthermore, in accord with missing formal career paths for becoming a professor, there is no agreement about the formal end of a postdoc qualification phase (Krempkow, 2016), which contributes the difficulty of determining the total number of early career academics. The influential Bundesbericht wissenschaftlicher Nachwuchs (BuWiN – Konsortium Bundesbericht Wissenschaftlicher Nachwuchs, 2021), e.g., opted for a rather arbitrary age limit of 45 years to identify “offspring” proper. In the following, a distinction is only made between doctoral and non-doctoral researchers.

In addition to ordinary academic staff, universities have created positions for teaching staff for special tasks (Lehrkräfte für besondere Aufgaben, LfbA). These must be considered separately in our calculation as they carry a much heavier teaching load. To account for further teaching duties, we also include honorary teachers (Lehrbeauftragte) in our calculations, who are not employed but usually work on the basis of honorary contracts with very low remuneration.

Salaries for all employees except professors are based on the collective agreement of the federal states (Tarifvertrag der Länder, TV-L) with the exception of the state of Hesse, which has similar agreements. However, Germany's universities are creatively adjusting individual salaries to their needs and budgets by extending part-time employment to staff who effectively work much more hours (Kuhnt et al., 2022, p. 68–70). While university statistics list the number of full-time and part-time positions—for all academic staff, we calculate that 55% of people are paid full-time, for LfbAs this proportion is 52%—, they do not list the average weekly working hours.

Concerning financial circumstances, salaries play a subordinate role compared to the sources of funding. Forty percent of all non-professorial academic positions are funded by project money (ICEland 60402)—usually for research projects—, although universities rely heavily on them to maintain their basic functions in research and sometimes even teaching. However, they are reluctant to create permanent positions from these budgets even in situations where the influx of funds is permanently renewed. Universities act under the presumption that project income could stop completely at any time. The situation is well illustrated by the fact that project funding accounts for a whole of only 1,365 permanent positions compared to 30,470 permanent positions funded by the universities' budgets.

Despite the frequently used term “third party funding” (Drittmittel), 85%^4^ of these funds come from public sources. It is therefore a political decision to organize funding in this way, further promoting fixed-term employment without clear career opportunities. Interestingly, even the German Science and Humanities Council (Wissenschaftsrat, 2023), convened by the federal government, advocates for reforms. Another reason for a lack of structured career opportunities at German universities is the atomized structure of decision-making mentioned in the beginning. Not only do professors “own” project-funded positions that are granted to them, but in most cases units with project-funded and budgetary positions combined are simply too small to allow for an employment structure that encompasses all career stages and thus could allow for steady upward mobility.

## 3 Developing models for university employment

In order to propose new employment structures for academic staff, available budgets and required teaching loads have to be estimated. To this end, we developed status quo models based on statistical data in this section. In Section 4, we will then present and discuss alternative models that overcome the current precarity, chains of fixed-term contracts and poor career perspectives for university staff. Our aim is, in other words, to develop models that can be compared to the status quo in terms of budget, teaching load, size of staff and transition rates.

### 3.1 Status quo

According to federal statistics (ICEland 60402), 113 German universities (not including universities of applied sciences, schools of education, art schools and theological universities) employed 25,645 full professors in 2021 (Table 1). This figure also includes “junior”-professors—which is only partially convincing regarding their employment situation. However, due to the small number of such positions, they are not listed separately here. Apart from professors, the universities employed 193,365 persons as academic staff plus 6,490 LfbAs. Of these, 114,225 (60%) staff members and 6,150 (97%) LfbAs have budgetary funding, the rest being project-funded. In the following, no further distinction is made among the LfbAs due to the low proportion of project-funded positions.

As already mentioned, temporary positions are the dominant form of employment, amounting to 82% of the positions of academic staff not holding a full professorship. Within this group, LfbAs are the only exception with a fixed-term rate of 30%. This can be explained, at least in part, by the tighter legal constraints for such contracts.

Information that allows to differentiate between employees with and without a doctoral degree is only available in aggregated form for all academic staff positions (ICEland 60302), for which we calculate a share of 36% of doctoral personnel. As a rough approximation to calculate a more detailed status quo model, we consider all permanent positions (budget: 30,470, project: 1,365) to be occupied by doctoral staff (Table 2), although our own survey data show that some (3.7%) of predoctoral employees also have a permanent post. The factor of fixed-term positions (47%) that are project-funded is assumed to be the same for both, predoctoral (budget: 64,317, project: 57,993) and doctoral researchers (budget: 19.438, project: 17.527). The contractually paid working hours of all positions are taken from the authors' own survey data and are shown in Tables 1–**9** (Kuhnt et al., 2022). A differentiation between budget and project funded positions was not necessary in this respect, as differences were not significant.

**Table 2:** Status quo model of all fields of science.

**Position**	**Count**	**Part-time- factor**	**FTE**	**Salary**	**Costs**	**Teaching duties**	**Total TD**	**Employment**
Professors	25,645	1.00	25,645	114,300 €	2,931,223,500 €	9	230,805	Permanent
Postdocs budget-funded permanent	30,470	0.93	28,337	82,569 €	2,515,886,147 €	8	226,697	Permanent
Postdocs budget-funded fixed-term	19,438	0.89	17,300	71,808 €	1,395,805,076 €	4	69,199	Fixed-term
Postdocs project-funded permanent	1,365	0.93	1,269	82,569 €	112,707,075 €	0	0	Permanent
Postdocs project-funded fixed-term	17,527	0.89	15,599	71,808 €	1,258,566,048 €	0	0	Fixed-term
Teaching staff (LfbA) permanent	4,555	0.85	3,872	75,467 €	343,750,143 €	16	61,948	Permanent
Teaching staff (LfbA) fixed-term	1,935	0.75	1,451	60,513 €	117,092,171 €	16	23,220	Fixed-term
Predocs budget-funded	64,317	0.78	50,167	53,516 €	3,441,964,825 €	4	200,669	Fixed-term
Predocs project-funded	57,993	0.78	45,235	53,516 €	3,103,542,279 €	0	0	Fixed-term
Honorary teaching contracts	29,765			1,000 €	29,765,000 €	2	59,530	
Total					15,250,302,264 €		872,068	

FTE, full time equivalents.

Large numbers, such as those shown in Table 2 for all German universities, are challenging to read, and are unsuited for part of our objective, since we are specifically interested in units such as departments instead of individual professorships and their subordinate staff. We have therefore projected the comprehensive statistical data to a model institute with six professors, in the following referred to as *six-professor institutes*, in order to make the status quo comparable to our model proposals (Table 3). Since the size of the model institutes has no influence on proportional distributions in the subsequent model evaluations, it suffices to make a plausible guess for an average institute size. All models can easily be scaled to other sizes. Minor difficulties arise where fractional numbers are to be avoided, but this will be discussed in more detail later.

**Table 3:** Status quo six professor institute for all fields of science.

**Position**	**Count**	**Part-time-factor**	**FTE**	**Salary**	**Costs**	**Teaching duties**	**Total TD**	**Employment**	**Duration (years)**	**Annually ending employments**	**New employments every … years**
Professors	6.0	1.00	6	114,300 €	685,800 €	9	54	Permanent	24.5	0.24	4.08
Postdocs budget-funded permanent	7.1	0.93	7	82,569 €	588,626 €	8	53	Permanent	25	0.29	3.51
Postdocs budget-funded fixed-term	4.5	0.89	4	71,808 €	326,568 €	4	16	Fixed-term	6	0.76	1.32
Postdocs project-funded permanent	0.3	0.93	0	82,569 €	26,369 €	0	0	Permanent	25	0.01	78.28
Postdocs project-funded fixed-term	4.1	0.89	4	71,808 €	294,459 €	0	0	Fixed-term	6	0.68	1.46
Teaching staff (LfbA) permanent	1.1	0.85	1	75,467 €	80,425 €	16	14	Permanent	30	0.04	28.15
Teaching staff (LfbA) fixed-term	0.5	0.75	0	60,513 €	27,395 €	16	5	Fixed-term	6	0.08	13.25
Predocs budget-funded	15.0	0.78	12	53,516 €	805,295 €	4	47	Fixed-term	6	2.51	0.40
Predocs project-funded	13.6	0.78	11	53,516 €	726,116 €	0	0	Fixed-term	6	2.26	0.44
Honorary teaching contracts	7.0			1,000 €	6,964 €	2	13.9				
Total					3,568,018 €		204.0				
**Model evaluation**	**Count**		**FTE**							**Annually**	
Staff without PhD	28.6		22.3				Turnover permanent employment			0.58	
Staff with PhD	23.6		21.9				Turnover fixed-term postdoc employment			1.52	
Staff total	52.2		44.2				Turnover predoc employment			4.77	
Budget-funded cost share	70.6%						**Transition rates**				
Fixed-term share of total	72.2%						Fixed-term postdoc to permanent			38.1%	
Fixed-term share of academic staff	81.6%						Predoc to fixed-term postdoc			31.8%	

FTE, full time equivalents.

Constructing an average model institute may not sufficiently take into account disciplinary differences. Therefore, we repeated the above steps for three exemplary scientific fields: law, economics and social sciences, mathematics and natural sciences, and engineering sciences; hereafter referred to as social sciences, natural sciences, and engineering, respectively. These three disciplinary fields were chosen because of their rather different staff compositions and thus employment costs, so that our models can cover the full variety of university employment. This also includes different project funding and postdoctoral employee ratios as well as paid weekly hours (Tables 4–**9**). The analysis of the total budget for different disciplines once again highlights the significant differences in funding, for example between engineering and social sciences, also in light of the fact that in 2021 50,915 students completed their studies in social sciences compared to 24,210 in engineering (ICEland 51301). This is an imbalance that should be discussed elsewhere.

**Table 4:** Status quo model of the social sciences.

**Position**	**Count**	**Part-time-factor**	**FTE**	**Salary**	**Costs**	**Teaching duties**	**Total TD**	**Employment**
Professors	6,150	1.00	6,150	114,300 €	702,945,000 €	9	55,350	Permanent
Postdocs budget-funded permanent	1,785	0.87	1,553	77,242 €	137,877,390 €	8	12,424	Permanent
Postdocs budget-funded fixed-term	2,604	0.88	2,291	71,002 €	184,876,792 €	8	18,331	Fixed-term
Postdocs project-funded permanent	130	0.87	113	77,242 €	10,041,491 €	0	0	Permanent
Postdocs project-funded fixed-term	1,424	0.88	1,253	71,002 €	101,075,159 €	0	0	Fixed-term
Teaching staff (LfbA) permanent	685	0.85	582	75,467 €	51,694,588 €	16	9,316	Permanent
Teaching staff (LfbA) fixed-term	650	0.75	488	60,513 €	39,333,288 €	16	7,800	Fixed-term
Predocs budget-funded	12,166	0.73	8,881	50,085 €	609,342,872 €	4	35,525	Fixed-term
Predocs project-funded	6,651	0.73	4,856	50,085 €	333,137,691 €	0	0	Fixed-term
Honorary teaching contracts	8,030			1,000 €	8,030,000 €	2	16,060	
Total					2,178,354,270 €		154,806	

FTE, full time equivalents.

### 3.2 Model constraints

The aim of our approach is it to identify the degrees of freedom for changing universities' employment structures without changing the budget and teaching capacities. Budgets are calculated by multiplying the number of people and the average paid working hours by an estimate of average personnel costs. The cost of a professor is directly adopted from the respective rates of the German research Foundation (DFG)^5^ and amounts to €114,300 annually. For all other positions, in order to take into account salaries that increase with the length of employment, the average employer costs were calculated based on the collective agreement of the federal states (Tarifvertrag der Länder, TVL) for every successive year of employment (cf. Supplementary Table 1). Three relevant employment phases are identified for the status quo: year 1 to 6 for predocs, year seven to 12 for postdocs as well as LfbA with fixed-term contracts, and year 13 to 40 for employees with permanent contracts. Assuming uniformly distributed times of employment we can then calculate average employment costs as shown in Tables 2–**17**.

Consequently, we arrive at an estimate of total staff expenses of German universities (Table 2). A comparative calculation for the three exemplary disciplines shows some differences between these fields with a total expenditure of €2.2 bn for the social sciences (Table 4), €2.8 bn for engineering (Table 5), and €2.9 bn for the natural sciences (Table 6). Against the background of these budgets, engineering employs in total only 3,810 professors compared to natural science with 5,690 and social science with 6,150 professors. This leads to higher budgets, and therefore more employees, per professor. Thus, budgets for the three disciplinary six-professor institutes amount to €2.1 mn (Table 7), €4.4 mn (Table 8), and €3.1 (Table 9) mn for social sciences, engineering and natural sciences, respectively.

**Table 5:** Status quo model of the engineering sciences.

**Position**	**Count**	**Part-time-factor**	**FTE**	**Salary**	**Costs**	**Teaching duties**	**Total TD**	**Employment**
Professors	3,810	1.00	3,810	114,300 €	435,483,000 €	9	34,290	Permanent
Postdocs budget-funded permanent	2,505	0.95	2,380	84,345 €	211,284,149 €	8	19,038	Permanent
Postdocs budget-funded fixed-term	1,056	0.95	1,004	76,649 €	80,980,034 €	4	4,015	Fixed-term
Postdocs project-funded permanent	605	0.95	575	84,345 €	51,028,707 €	0	0	Permanent
Postdocs project-funded fixed-term	2,111	0.95	2,005	76,649 €	161,807,204 €	0	0	Fixed-term
Teaching staff (LfbA) permanent	175	0.85	149	75,467 €	13,206,647 €	16	2,380	Permanent
Teaching staff (LfbA) fixed-term	50	0.75	38	60,513 €	3,025,638 €	16	600	Fixed-term
Predocs budget-funded	9,539	0.93	8,871	63,807 €	608,623,095 €	4	35,483	Fixed-term
Predocs project-funded	19,059	0.93	17,725	63,807 €	1,216,097,302 €	0	0	Fixed-term
Honorary teaching contracts	2,385			1,000 €	2,385,000 €	2	4,770	
Total					2,783,920,775 €		100,576	

FTE, full time equivalents.

**Table 6:** Status quo model of the natural sciences.

**Position**	**Count**	**Part-time-factor**	**FTE**	**Salary**	**Costs**	**Teaching duties**	**Total TD**	**Employment**
Professors	5,690	1.00	5,690	114,300 €	650,367,000	9	51,210.0	Permanent
Postdocs budget-funded permanent	4,300	0.95	4,085	84,345 €	362,683,369	8	32,680.0	Permanent
Postdocs budget-funded fixed-term	3,071	0.90	2,764	72,615 €	222,990,953	4	11,055.1	Fixed-term
Postdocs project-funded permanent	1,010	0.95	960	84,345 €	85,188,419	0	0.0	Permanent
Postdocs project-funded fixed-term	4,597	0.90	4,137	72,615 €	333,808,645	0	0.0	Fixed-term
Teaching staff (LfbA) permanent	420	0.85	357	75,467 €	31,695,952	16	5,712.0	Permanent
Teaching staff (LfbA) fixed-term	235	0.75	176	60,513 €	14,220,496	16	2,820.0	Fixed-term
Predocs budget-funded	10,089	0.70	7,062	48,027 €	484,549,082	4	28,249.6	Fixed-term
Predocs project-funded	15,103	0.70	10,572	48,027 €	725,350,829	0	0.0	Fixed-term
Honorary teaching contracts	2,050.0			1,000 €	2,050,000 €	2	4,100.0	
Total					2,912,904,745 €		135,826.7	

FTE, full time equivalents.

**Table 7:** Status quo six professor institute for the social sciences.

**Position**	**Count**	**Part-time-factor**	**FTE**	**Salary**	**Costs**	**Teaching duties**	**Total TD**	**Employment**	**Duration (years)**	**Annually ending employments**	**New employments every … years**
Professors	6.0	1.00	6.0	114,300 €	685,800 €	9	54.0	Permanent	24,5	0.24	4.08
Postdocs budget-funded permanent	1.7	0.87	1.5	77,242 €	134,515 €	8	12.1	Permanent	25	0.07	14.36
Postdocs budget-funded fixed-term	2.5	0.88	2.2	71,002 €	180,368 €	4	8.9	Fixed-term	6	0.42	2.36
Postdocs project-funded permanent	0.1	0.87	0.1	77,242 €	9,797 €	0	0.0	Permanent	25	0.01	197.12
Postdocs project-funded fixed-term	1.4	0.88	1.2	71,002 €	98,610 €	0	0.0	Fixed-term	6	0.23	4.32
Teaching staff (LfbA) permanent	0.7	0.85	0.6	75,467 €	50,434 €	16	9.1	Permanent	30	0.02	44.89
Teaching staff (LfbA) fixed-term	0.6	0.75	0.5	60,513 €	38,374 €	16	7.6	Fixed-term	6	0.11	9.46
Predocs budget-funded	11.9	0.73	8.7	50,085 €	594,481 €	4	34.7	Fixed-term	6	1.98	0.51
Predocs project-funded	6.5	0.73	4.7	50,085 €	325,012 €	0	0.0	Fixed-term	6	1.08	0.92
Honorary teaching contracts	7.8			1,000 €	7,834 €	2	15.7				
Total					2,125,224 €		142.1				
**Model evaluation**	**Count**		**FTE**							**Annually**	
Staff without PhD	18.4		13.4				Turnover permanent employment			0.34	
Staff with PhD	13.1		12.1				Turnover fixed-term postdoc employment			0.76	
Staff total	31.5		25.5				Turnover predoc employment			3.06	
Budget-funded cost share	79.5%						**Transition rates**				
Fixed-term share of total	72.9%						Fixed-term postdoc to permanent			45.0%	
Fixed-term share of academic staff	90.0%						Predoc to fixed-term postdoc			24.9%	

FTE, full time equivalents.

**Table 8:** Status quo six professor institute for the engineering sciences.

**Position**	**Count**	**Part-time-factor**	**FTE**	**Salary**	**Costs**	**Teaching duties**	**Total TD**	**Employment**	**Duration (years)**	**Annually ending employments**	**New employments every … years**
Professors	6.0	1.00	6	114,300 €	685,800 €	9	54.0	Permanent	24.5	0.24	4.08
Postdocs budget-funded permanent	3.9	0.95	4	84,345 €	332,731 €	8	30.0	Permanent	25	0.16	6.34
Postdocs budget-funded fixed-term	1.7	0.95	2	76,649 €	127,528 €	4	6.3	Fixed-term	6	0.28	3.61
Postdocs project-funded permanent	1.0	0.95	1	84,345 €	80,360 €	0	0.0	Permanent	25	0.04	26.24
Postdocs project-funded fixed-term	3.3	0.95	3	76,649 €	254,814 €	0	0.0	Fixed-term	6	0.55	1.80
Teaching staff (LfbA) permanent	0.3	0.85	0	75,467 €	20,798 €	16	3.7	Permanent	30	0.01	108.86
Teaching staff (LfbA) fixed-term	0.1	0.75	0	60,513 €	4,765 €	16	0.9	Fixed-term	6	0.01	76.20
Predocs budget-funded	15.0	0.93	14	63,807 €	958,462 €	4	55.9	Fixed-term	6	2.50	0.40
Predocs project-funded	30.0	0.93	28	63,807 €	1,915,114 €	0	0.0	Fixed-term	6	5.00	0.20
Honorary teaching contracts	3.8			1,000 €	3,756 €	2	7.5				
Total					4,384,127 €		158.4				
**Model evaluation**	**Count**		**FTE**							**Annually**	
Staff without PhD	45.0		41.9				Turnover permanent employment			0.45	
Staff with PhD	16.2		15.7				Turnover fixed-term postdoc employment			0.84	
Staff total	61.3		57.6				Turnover predoc employment			7.51	
Budget-funded cost share	48.6%						**Transition rates**				
Fixed-term share of total	81.8%						Fixed-term postdoc to permanent			53.3%	
Fixed-term share of academic staff	90.6%						Predoc to fixed-term postdoc			11.3%	

FTE, full time equivalents.

**Table 9:** Status quo six professor institute for the natural sciences.

**Position**	**Count**	**Part-time-factor**	**FTE**	**Salary**	**Costs**	**Teaching duties**	**Total TD**	**Employment**	**Duration (years)**	**Annually ending employments**	**New employments every … years**
Professors	6.0	1.00	6	114,300 €	685,800 €	9	54.0	Permanent	25.5	0.24	4.25
Postdocs budget-funded permanent	4.5	0.95	4	84,345 €	382,443 €	8	34.5	Permanent	25	0.18	5.51
Postdocs budget-funded fixed-term	3.2	0.90	3	72,615 €	235,140 €	4	11.7	Fixed-term	6	0.54	1.85
Postdocs project-funded permanent	1.1	0.95	1	84,345 €	89,830 €	0	0.0	Permanent	25	0.04	23.47
Postdocs project-funded fixed-term	4.8	0.90	4	72,615 €	351,995 €	0	0.0	Fixed-term	6	0.81	1.24
Teaching staff (LfbA) permanent	0.4	0.85	0	75,467 €	33,423 €	16	6.0	Permanent	30	0.01	67.74
Teaching staff (LfbA) fixed-term	0.2	0.75	0	60,513 €	14,995 €	16	3.0	Fixed-term	6	0.04	24.21
Predocs budget-funded	10.6	0.70	7	48,027 €	510,948 €	4	29.8	Fixed-term	6	1.77	0.56
Predocs project-funded	15.9	0.70	11	48,027 €	764,869 €	0	0.0	Fixed-term	6	2.65	0.38
Honorary teaching contracts	2.2			1,000 €	2,162 €	2	4.3				
Total					3,071,604 €		143.2				
**Model evaluation**	**Count**		**FTE**							**Annually**	
Staff without PhD	26.6		18.6				Turnover permanent employment			0.47	
Staff with PhD	20.4		19.2				Turnover fixed-term postdoc employment			1.39	
Staff total	46.9		37.8				Turnover predoc employment			4.43	
Budget-funded cost share	60.7%						**Transition rates**				
Fixed-term share of total	74.3%						Fixed-term postdoc to permanent			34.1%	
Fixed-term share of academic staff	85.2%						Predoc to fixed-term postdoc			31.4%	

FTE, full time equivalents.

The teaching quotas for the institutes were once more calculated by multiplying average paid working hours with typical individual teaching duties on specific positions. These teaching duties are regulated at state level but generally differ only slightly between the states, with the sole exception of Brandenburg which in principle allows agreements of up to 24 teaching hours per week for all positions. Under our assumptions, the six-professor institutes would thus have to provide 204 weekly teaching hours over all disciplines (Table 3), and due to disciplinary differences 142 h in social sciences, 158 h in engineering and 143 in natural sciences.^6^

### 3.3 Model evaluation

The status quo six-professor institute employs a total of 52 people, which corresponds to 44 full-time equivalents. Of these employees, including professors, 72% have a fixed-term contract, and 55% do not hold a doctoral degree. The social science institute is much smaller with only 31 employees, or 26 full-time equivalents, with a fixed-term rate of 73 and 58% persons not holding a PhD. As already mentioned, an engineering institute is structured very differently, with 61 persons or 58 full time equivalents, a fixed-term rate of 82% and a predoctoral share of 74%. Natural sciences find themselves between the others with 47 persons, 38 full-time equivalents, a fixed-term proportion of 74 and 57% of employees not holding a PhD.^7^

Central to our argument, however, are the transition rates between different career phases. To calculate these, we assume an average duration of contracts for each employment phase, since deviations from the mean will cancel each other out (Table 7). Our calculation also takes the 6-year periods of predoc and postdoc employment as a whole, omitting that after a period of 6 years of fixed-term employment, employees look back on about four consecutive contracts if an average contract duration of 20 months is assumed (Sommer et al., 2022, p. XI).

According to official statistics, students complete their diploma or master's degree or equivalent at an average age of 26.6 years (ICEland 34501). A doctoral examination is taken at an average age of 32.2 years. The difference of 5.6 years neatly corresponds to the current 6 year limit of fixed-term employment for doctoral students. Further statistical information about typical career paths is unfortunately scarce. It must be taken into account that some doctoral students never complete their doctoral thesis and drop out earlier. At the same time, there are exceptions that allow for continued fixed-term employment at a university even without a doctorate. We therefore assume that these effects cancel each other out and a predoc period of 6 years can be assumed. A 6-year duration of the predoc-phase was kept constant for all proposed models. Similar considerations underlie our assumptions for the postdoc phase. Here too, fixed-term employment on budgetary positions is permitted for a further 6 years and universities tend to exhaust these possibilities. At the same time, it is to be expected that some of the staff leave the university earlier while others remain in fixed-term employment contracts even after a 6-year postdoc period, namely on project-funded positions.

At a later stage of the postdoc phase, different employment relationships may coexist: some stay somewhat longer in fixed-term postdoc positions before leaving the system or taking up a professorship. Others take up a teaching position as LfbA which in the majority of cases is a permanent position. We assume that the latter are taken at a somewhat earlier stage of employment in academia and are therefore held for an average of 30 years. The situation is similar for permanent positions as academic staff, although here we assume that such positions are held for 25 years due to a later start. Statistical data allowing to estimate the duration of professorial employment is much better. The beginning of a professorship is statistically taking place at the average age of 42.5 years (BuWiN – Konsortium Bundesbericht Wissenschaftlicher Nachwuchs, 2021, p. 91, estimated for W2 and W3 positions), leaving 24.5 years until retirement.

With this information, we can calculate and compare for various models how many people finish a particular stage of their academic career and, accordingly, how many positions at the next stage become vacant at the same time. Taking our six-professor institute, 4.8 predoc positions become vacant each year and can be taken by new doctoral students. For these 4.8 people who now hold a doctoral degree, 1.5 postdoc positions are available, which corresponds to a transition rate of 32%. At the same time, 1.5 postdoctoral employees reach the end of a fixed-term employment each year. Of these, only 0.6 will find a vacant permanent position. At a relatively advanced age, therefore, only 38% of employees can continue working in academia. Figures differ significantly for different disciplines in our model calculation, with a transition rate after completing a doctorate of 25% in the social sciences, 11% in engineering and 31% in the natural sciences. The success rate in achieving a permanent position after the postdoc phase is 45% in social sciences, 53% in engineering and 34% in the natural sciences.

## 4 Alternative models

As alternatives to this unsatisfactory status quo, we propose a *tenure-track* and a *lecturer model*. These two approaches do not completely abolish the idea of selection in the course of an academic career but create organized, fair and sustainable ways of attaining positions, with the main exit option situated directly after finishing a dissertation. The aim is to overcome the problematic combination of short-term fixed contracts and a great uncertainty about the possibility of continued employment at a German university, and to ensure the recruitment of professionalized academic personnel. This proposed reform also involves a shift from providing basic academic functions by personnel without a doctorate to the employment of more permanent staff, with almost no change in overall staff numbers.

To improve doctoral students' readiness for academic careers, fundamental reforms of graduate programs are required. Our proposal aims at prioritizing doctoral students' career development over fulfilling universities' requirements by reducing the teaching loads for doctoral students to just one semester hour per week (SWS), equating to one course annually. This adjustment allows students to improve their teaching abilities without the pressure of rushing through course preparation and inadvertently sidelining their research pursuits and responsibilities.

Project-funded positions are—at least in theory—exempt from any teaching obligations, which leads to quite problematic imbalances when it is highly contingent whether a position is budget- or project-funded. In our models, we do not calculate with a complete and radical change, although, in accordance with current discussions, we propose a lower project-funding rate. Furthermore, we introduce the additional criterium that experienced teachers will be available at the postdoc level. Therefore, our models ensure that the rate of budget-funded doctoral students that finish their doctorate every year always exceeds free postdoc positions.

Throughout all disciplines, the proposed models increase the factor of paid weekly hours for doctoral students to 0.8, except for engineering with a factor of 0.9. This constitutes a compromise since full positions would be desirable. However, since the aim of the proposed models is to show how to improve the status quo, we reduced but not removed the problem of possible underpayment on the predoctoral level. A further transition to full positions would be feasible within teaching and budget constraints. Such a transition would reduce the total number of employed doctoral students which, in turn, would imply slightly smaller chances for students to achieve a doctoral position but higher transition rates toward a postdoc position.

We also reduced the number of external teaching contractors from the current average of seven to only two in our models. Such contracts may have some legitimacy if external experts can actually be hired to offer courses to complement the regular curriculum. However, in many cases, this option is abused by universities to hire cheap personnel for providing the core curriculum.

Even though tenure-track positions play only a minor role in German academic employment today, an expansion of this category is a focus in the debate and an objective of the policy of the Federal Ministry for Education and Science (BMBF). Our first model will thus calculate how a system with tenure track as the regular career path could look like. Although such a system is feasible and clearly superior to the status quo in plannability and transparency, we will also encounter modeling difficulties and practical doubts. The selection process before gaining a full professorship poses the problem of how to provide the adequate number of positions, institutes will become relatively small, and in some versions, the teaching load will only just be met. To look for potentially superior alternatives, we then propose a lecturer model that involves only one selection stage after attaining a doctoral degree.

### 4.1 Tenure track

A consistent implementation of a tenure-track system to prepare for a professorship requires a significant increase of professorial positions, similar to what has already been proposed by the Young Academy (Junge Akademie), a German group of elite early career researchers (Specht et al., 2017). As can be seen in Table 10, such a model is feasible. In all our tenure-track models, once more differentiated for disciplines (Tables 11–**13**), we choose the number of professors vs. tenure-track position holders in such a way that a success rate of about 75% would be possible, meaning that three out of four tenure track candidates could gain a full professorship position after a period of 6 years. The idea here is not that the success rate should be effectively capped, but that a system moving around such success rates would work. For those tenure-track holders who are unsuccessful in their evaluation after 4 years we took into account the current practice of allowing some time for finishing publications and finding a new employment before leaving the university. In our models, we set this transitional employment period to 2 years, providing for 6 years in total regardless of whether an evaluation was successful or not. After successful completion of the tenure-track phase, the start of a professorship is assumed at a much earlier point than today, at an age of 32 years. The period until retirement is therefore extended to 35 years.

**Table 10:** Tenure-track department for all fields of science.

**Position**	**Count**	**Part-time-factor**	**FTE**	**Salary**	**Costs**	**Teaching duties**	**Total TD**	**Employment**	**Duration (years)**	**Annually ending employments**	**New employments every … years**
Professors	19.0	1.00	19	114,300 €	2,171,700 €	9	171.0	Permanent	35	0.54	1.84
Tenure-Track	4.0	1.00	4	80,684 €	322,735 €	5	20.0	Fixed-term	6	0.67	1.50
Predocs budget-funded	13.0	0.80	10	54,888 €	713,541 €	1.25	13.0	Fixed-term	6	2.17	0.46
Predocs project-funded	6.0	0.80	5	54,888 €	329,326 €	0	0.0	Fixed-term	6	1.00	1.00
Honorary teaching contracts	2			1,500 €	3,000 €	2	4.0				
Total					3,540,302 €		208.0				
**Model evaluation**	**Count**		**FTE**							**Annually**	
Staff without PhD	19.0		15.2				Turnover permanent employment			0.54	
Staff with PhD	23.0		23.0				Turnover fixed-term postdoc employment			0.67	
Staff total	42.0		38.2				Turnover predoc employment			3.17	
Budget-funded cost share	90.7%						**Transition rates**				
Fixed-term share of total	54.8%						Fixed-term postdoc to permanent			81.4%	
Fixed-term share of academic staff	100.0%						Predoc to fixed-term postdoc			21.1%	

FTE, full time equivalents.

**Table 11:** Tenure-track department for the social sciences.

**Position**	**Count**	**Part-time-factor**	**FTE**	**Salary**	**Costs**	**Teaching duties**	**Total TD**	**Employment**	**Duration (years)**	**Annually ending employments**	**New employments every … years**
Professors	13.0	1.00	13	114,300 €	1,485,900 €	9	117.0	Permanent	35	0.37	2.69
Tenure-Track	3.0	1.00	3	88,784 €	266,353 €	5	15.0	Fixed-term	6	0.50	2.00
Predocs budget-funded	5.0	0.80	4	54,888 €	274,439 €	1.25	5.0	Fixed-term	6	0.83	1.20
Predocs project-funded	2.0	0.80	2	54,888 €	109,775 €	0	0.0	Fixed-term	6	0.33	3.00
Honorary teaching contracts	2			1,500 €	3,000 €	2	4.0				
Total					2,139,467 €		141.0				
**Model evaluation**	**Count**		**FTE**							**Annually**	
Staff without PhD	7.0		5.6				Turnover permanent employment			0.37	
Staff with PhD	16.0		16.0				Turnover fixed-term postdoc employment			0.50	
Staff total	23.0		21.6				Turnover predoc employment			1.17	
Budget-funded cost share	94.9%						**Transition rates**				
Fixed-term share of total	43.5%						Fixed-term postdoc to permanent			74.3%	
Fixed-term share of academic staff	100.0%						Predoc to fixed-term postdoc			42.9%	

FTE, full time equivalents.

In this constellation, the department would need 19 full professors and four tenure-track positions to meet teaching requirements (Table 10). For the ease of the argument, professorial salary was not reduced compared to the status quo, even though such positions are taken up 10 years earlier in the career. The given budget then allows for a total of 19 doctoral students.

The relatively low teaching obligations on tenure-track positions become a serious constraint in the case of the social science department with a relatively small budget (Table 11). To meet teaching requirements, such an institute would have to accommodate 13 full professors and three tenure-track positions. The given budget would only allow for the additional employment of seven doctoral students in total. The situation is somewhat more relaxed for the institutes of the other two disciplinary cultures. In the engineering department, there would be room for 22 full professorships, five tenure-track positions and a total of 23 doctoral students (Table 12). For the natural sciences department we had to resort to fractional numbers, which we so far avoided, and calculate 16 full professors and 3.5 tenure-track positions (Table 13). The fractional numbers can, e.g., be realized by alternating between three and four positions in consecutive terms or by adjusting the department size. Such a model department could accommodate 17 doctoral students.

**Table 12:** Tenure-track department for the engineering sciences.

**Position**	**Count**	**Part-time-factor**	**FTE**	**Salary**	**Costs**	**Teaching duties**	**Total TD**	**Employment**	**Duration (years)**	**Annually ending employments**	**New employments every … years**
Professors	22.0	1.00	22	114,300 €	2,514,600 €	9	198.0	Permanent	35	0.63	1.59
Tenure-Track	5.0	1.00	5	88,784 €	443,921 €	5	25.0	Fixed-term	6	0.83	1.20
Predocs budget-funded	10.0	0.90	9	61,749 €	617,487 €	1,11	10.0	Fixed-term	6	1.67	0.60
Predocs project-funded	13.0	0.90	12	61,749 €	802,733 €	0	0.0	Fixed-term	6	2.17	0.46
Honorary teaching contracts	2			1,500 €	3,000 €	2	4.0				
Total					4,381,741 €		237.0				
**Model evaluation**	**Count**		**FTE**							**Annually**	
Staff without PhD	23.0		20.7				Turnover permanent employment			0.63	
Staff with PhD	27.0		27.0				Turnover fixed-term postdoc employment			0.83	
Staff total	50.0		47.7				Turnover predoc employment			3.83	
Budget-funded cost share	81.7%						**Transition rates**				
Fixed-term share of total	56.0%						Fixed-term postdoc to permanent			75.4%	
Fixed-term share of academic staff	100.0%						Predoc to fixed-term postdoc			21.7%	

FTE, full time equivalents.

**Table 13:** Tenure-track department for the natural sciences.

**Position**	**Count**	**Part-time-factor**	**FTE**	**Salary**	**Costs**	**Teaching duties**	**Total TD**	**Employment**	**Duration (years)**	**Annually ending employments**	**New employments every … years**
Professors	16.0	1.00	16	114,300€	1,828,800€	9	144.0	Permanent	35	0.46	2.19
Tenure-Track	3.5	1.00	4	88,784€	310,745€	5	17.5	Fixed-term	6	0.58	1.71
Predocs budget-funded	11.0	0.80	9	54,888€	603,765€	1.25	11.0	Fixed-term	6	1.83	0.55
Predocs project-funded	6.0	0.80	5	54,888€	329,326€	0	0.0	Fixed-term	6	1.00	1.00
Honorary teaching contracts	2			1,500€	3,000€	2	4.0				
total					3,075,636€		176.5				
**Model evaluation**	**Count**		**FTE**							**Annually**	
Staff without PhD	17.0		13.6				Turnover permanent employment			0.46	
Staff with PhD	19.5		19.5				Turnover fixed-term postdoc employment			0.58	
Staff total	36.5		33.1				Turnover predoc employment			2.83	
Budget-funded cost share	89.3%						**Transition rates**				
Fixed-term share of total	56.2%						Fixed-term postdoc to permanent			78.4%	
Fixed-term share of academic staff	100.0%						Predoc to fixed-term postdoc			20.6%	

FTE, full time equivalents.

### 4.2 Lecturers

The lecturer model follows the principle that by achieving a doctoral degree, a researcher should have proven her or his qualification to work as a scientist. At this point, a university must make a decision about employing persons on a permanent position. By suggesting this model, we aim to demonstrate that scientific careers can be organized without being confronted with existential threats for long time spans.

The size of the exemplary department is chosen once more to accommodate six professors. The available budget then determines the total number of lecturers and doctoral students. The lecturer model allows for much higher degrees of freedom in choosing the number of lecturers and doctoral students, as constraints in teaching output are easily met. In the general model, a total number of 20.5 lecturers and of 20 doctoral students was chosen (Table 14). The 20.5 lecturers are divided into those two to three persons who take up a professorship after 10 years and those who do not and therefore remain on these positions presumably until retirement after 35 years. As before, the fractional numbers used here can be realized by alternating numbers, by various time frames or different department sizes. It should be noted that even whole numbers only represent averages as well.

**Table 14:** Lecturer department for all fields of science.

**Position**	**Count**	**Part-time-factor**	**FTE**	**Salary**	**Costs**	**Teaching duties**	**Total TD**	**Employment**	**Duration (years)**	**Annually ending employments**	**New employments every … years**
Professors	6.0	1.00	6	114,300€	685,800€	9	54.0	Permanent	24.5	0.24	4.08
Lecturer	18.0	1.00	18	87,355€	1,572,384€	8	144.0	Permanent	35	0.51	1.94
Lecturer taking up professurship with 42 years	2.5	1.00	3	83,300€	208,251€	8	20.0	Permanent	10	0.25	4.00
Predocs budget-funded	14.0	0.80	11	54,888€	768,428€	1,25	14.0	Fixed-term	6	2.33	0.43
Predocs project-funded	6.0	0.80	5	54,888€	329,326€	0	0.0	Fixed-term	6	1.00	1.00
Honorary teaching contracts	2			1,500€	3,000€	2	4.0				
Total					3,567,190€		236.0				
**Model evaluation**	**Count**		**FTE**							**Annually**	
Staff without PhD	20.0		16.0				Turnover permanent employment			0.76	
Staff with PhD	26.5		26.5				Turnover fixed-term postdoc employment			–	
Staff total	46.5		42.5				Turnover predoc employment			3.33	
Budget-funded cost share	84.9%						**Transition rates**				
Fixed-term share of total	43.0%						Fixed-term postdoc to permanent			–	
Fixed-term share of academic staff	49.4%						Predoc to fixed-term postdoc			22.9%	

FTE, full time equivalents.

In the following models, we adjusted the ratio of lecturers and doctoral students and thus the transition rates to a certain degree to take into account different disciplinary budgets and cultures. As a result, in addition to the six professors, the social science department accommodates 11 lecturers and nine doctoral students (Table 15), the engineering department 22.5 lecturers and 28 doctoral students (Table 16), and the natural science department 17.5 lecturers and 16 doctoral students (Table 17). For a better adjustment of our models, we resorted to fractural numbers again.

**Table 15:** Lecturer department for the social sciences.

**Position**	**Count**	**Part-time-factor**	**FTE**	**Salary**	**Costs**	**Teaching duties**	**Total TD**	**Employment**	**Duration (years)**	**Annually ending employments**	**New employments every … years**
Professors	6.0	1.00	6	114,300 €	685,800 €	9	54.0	Permanent	24.5	0.24	4.08
Lecturer	8.5	1.00	9	88,784 €	754,666 €	8	68.0	Permanent	35	0.24	4.12
Lecturer taking up professurship with 42 years	2.5	1.00	3	83,300 €	208,251 €	8	20.0	Permanent	10	0.25	4.00
Predocs budget-funded	6.0	0.80	5	54,888 €	329,326 €	1.25	6.0	Fixed-term	6	1.00	1.00
Predocs project-funded	3.0	0.80	2	54,888 €	164,663 €	0	0.0	Fixed-term	6	0.50	2.00
Honorary teaching contracts	2			1,500 €	3,000 €	2	4.0				
Total					2,145,706€		152.0				
**Model evaluation**	**Count**		**FTE**							**Annually**	
Staff without PhD	9.0		7.2				Turnover permanent employment			0.49	
Staff with PhD	17.0		17.0				Turnover fixed-term postdoc employment			–	
Staff total	26.0		24.2				Turnover predoc employment			1.50	
Budget-funded cost share	82.6%						**Transition rates**				
Fixed-term share of total	34.6%						Fixed-term postdoc to permanent			–	
Fixed-term share of academic staff	45.0%						Predoc to fixed-term postdoc			32.9%	

FTE, full time equivalents.

**Table 16:** Lecturer department for the engineering sciences.

**Position**	**Count**	**Part-time-factor**	**FTE**	**Salary**	**Costs**	**Teaching duties**	**Total TD**	**Employment**	**Duration (years)**	**Annually ending employments**	**New employments every … years**
Professors	6.0	1.00	6	114.300 €	685.800 €	9	54.0	Permanent	24.5	0.24	4.08
Lecturer	20.0	1.00	20	87.355 €	1.747.094 €	8	160.0	Permanent	35	0.57	1.75
Lecturer taking up professurship with 42 years	2.5	1.00	3	83.300 €	208.251 €	8	20.0	Permanent	10	0.25	4.00
Predocs budget-funded	14.0	0.90	13	61.749 €	864.482 €	1.11	14.0	Fixed-term	6	2.33	0.43
Predocs project-funded	14.0	0.90	13	61.749 €	864.482 €	0	0.0	Fixed-term	6	2.33	0.43
Honorary teaching contracts	2			1.500 €	3.000 €	2	4.0				
Total					4.373.108 €		252.0				
**Model evaluation**	**Count**		**FTE**							**Annually**	
Staff without PhD	28.0		25.2				Turnover permanent employment			0.82	
Staff with PhD	28.5		28.5				Turnover fixed-term postdoc employment			–	
Staff total	56.5		53.7				Turnover predoc employment			4.67	
Budget-funded cost share	75.5%						**Transition rates**				
Fixed-term share of total	49.6%						Fixed-term postdoc to permanent			–	
Fixed-term share of academic staff	55.4%						Predoc to fixed-term postdoc			17.6%	

FTE, full time equivalents.

**Table 17:** Lecturer department for the natural sciences.

**Position**	**Count**	**Part-time-factor**	**FTE**	**Salary**	**Costs**	**Teaching duties**	**Total TD**	**Employment**	**Duration (years)**	**Annually ending employments**	**New employments every … years**
Professors	6.0	1.00	6	114,300 €	685,800 €	9	54.0	Permanent	25.5	0.24	4.25
Lecturer	15.0	1.00	15	87,355 €	1,310,320 €	8	120.0	Permanent	35	0.43	2.33
Lecturer taking up professurship with 42 years	2.5	1.00	3	83,300 €	208,251 €	8	20.0	Permanent	10	0.25	4.00
Predocs budget-funded	10.0	0.80	8	54,888 €	548,877 €	1.25	10.0	Fixed-term	6	1.67	0.60
Predocs project-funded	6.0	0.80	5	54,888 €	329,326 €	0	0.0	Fixed-term	6	1.00	1.00
Honorary teaching contracts	2			1,500€	3,000€	2	4.0				
Total					3,085,575€		208.0				
**Model evaluation**	**Count**		**FTE**							**Annually**	
Staff without PhD	16.0		12.8				Turnover permanent employment			0.68	
Staff with PhD	23.5		23.5				Turnover fixed-term postdoc employment			–	
Staff total	39.5		36.3				Turnover predoc employment			2.67	
Budget-funded cost share	82.6%						**Transition rates**				
Fixed-term share of total	40.5%						Fixed-term postdoc to permanent			–	
Fixed-term share of academic staff	47.8%						Predoc to fixed-term postdoc			25.4%	

FTE, full time equivalents.

### 4.3 Total number of employees

The main concern of disputes about a sustainable system of academic employment should be not only to preserve but also to improve the performance of German universities with regard to research and teaching. Reliable employment and career structures are a fundamental instrument for this. An additional goal is to enable as many people as possible to work in science in order to preserve a variety of individual perspectives and social backgrounds. The models we propose do just this by increasing the number of people with a perspective of continued employment in the academic sector. While the status quo allows such a permanent perspective for only 14.5 people in the model institute, sometimes after a long period of uncertainty, the tenure-track model increases this number to 19 persons much earlier in their career, and the lecturer model to 26.5 persons, even earlier.

This can be achieved at the cost of only a slight reduction of the total number of employees. An additional decision built into our alternative models is to only include full-time or nearly full-time positions. Therefore, we also compared full-time equivalents: while the status quo model accommodates a total of 44 full-time equivalents, the tenure-track model has only 38. The lecturer model department with 43 people only demands a very small reduction compared to the status quo.

With regard to disciplinary variation, the social sciences department shows a very similar decline from 26 members in the status quo to 22 in the tenure-track and to 24 in the lecturer model. In engineering, the number drops from 58 to 48 or to 54, and for the natural sciences from 38 to 33 or to 36.

### 4.4 Project funding

The status quo ratio between the total number of staff financed from university budgets and those financed from projects represents a constraint that can be easily met in different versions of our lecturer models, although not always by the tenure-track models if the condition of a minimum number of budgetary doctoral students is maintained to ensure continuity. In the versions laid out here, we propose what we believe to be a healthier ratio with relatively less project funding, because this funding, due to its sometimes volatile nature, makes sustainable human resources planning difficult.

To further account for these imponderables, all departments in our alternative models direct project funding exclusively to the employment of doctoral students. Thus, even if status quo rates of project funding remain unchanged, a sufficient number of doctoral students would be available for later career stages in the unlikely case that all project funding should cease.

The increased rate of budget funded positions in our alternative models can be represented most clearly when we take into account all positions, including professorial positions. As we already mentioned, the overall share of budget funded positions under status quo conditions is 71%. Among the disciplines, engineering institutes are the most relying on project funding with a budget-funded share of 49%, followed by natural sciences with 61%, and the social sciences with 80%.

Since the number of doctoral positions in the tenure-track model is much more limited and we are trying to maintain a reasonable number of budget-funded doctoral positions, the proposed budget share in these models is quite high: 91% for the general model, 95% for the social sciences, 76% for the engineering and 89% for the natural sciences.

In the construction of lecturer departments, which allow for much higher degrees of variation, we propose 86% for the general model, 85% for the social science, 76% for the engineering and 83% for the natural science department. But even an engineering lecturer department with a budget share of 52% could still employ enough budget-funded doctoral students to ensure sufficient qualified teaching staff at the lecturer and professorial level (Table 18).

**Table 18:** Lecturer department for the engineering sciences with a current project-funding rate.

**Position**	**Count**	**Part-time-factor**	**FTE**	**Salary**	**Costs**	**Teaching duties**	**Total TD**	**Employment**	**Duration (years)**	**Annually ending employments**	**New employments every … years**
Professors	6.0	1.00	6	114,300 €	685,800 €	9	54.0	permanent	24.5	0.24	4.08
Lecturer	12.5	1.00	20	87,355 €	1,091,933 €	8	100.0	permanent	35	0.36	2.80
Lecturer taking up professurship with 42 years	2.5	1.00	3	83,300 €	208,251 €	8	20.0	permanent	10	0.25	4.00
Predocs budget-funded	6.0	0.90	13	61,749 €	370,492 €	1.11	6.0	fixed-term	6	1.00	1.00
Predocs project-funded	33.0	0.90	13	61,749 €	2,037,707 €	0	0.0	fixed-term	6	5.50	0.18
Honorary teaching contracts	2			1,500 €	3,000 €	2	4.0				
Total					4,397,184€		184.0				
**Model evaluation**	**Count**		**FTE**							**Annually**	
Staff without PhD	28.0		25.2				Turnover permanent employment			0.61	
Staff with PhD	28.5		28.5				Turnover fixed-term postdoc employment			–	
Staff total	56.5		53.7				Turnover predoc employment			6.50	
Budget-funded cost share	75.9%						**Transition rates**				
Fixed-term share of total	49.6%						Fixed-term postdoc to permanent			–	
Fixed-term share of academic staff	55.4%						predoc to fixed-term postdoc			9.3%	

FTE, full time equivalents.

### 4.5 Transition and temporary employment rates

The aim of the proposed models was to design career paths that include not only transparent and well-designed selection procedures but also fair transition chances from one career stage to the next. Under current conditions the probability of reaching a permanent position as a full professor, research associate or LfbA is 12% when multiplying the probabilities corresponding to the two hurdles to pass: a transition rate of 32% between pre- and a postdoc phase and—more problematic—a success rate of only 38% for reaching a permanent position at some point during the postdoc phase (Table 3).

It is therefore crucial that our alternative models imply a much earlier decision about whether a person stays at the university. Among these models, the tenure-track model proposed here (cf. Table 10) selects much stronger and from fewer people after a doctoral degree is awarded. The transition rate at this point is 21%. At a later stage, when scientists have already reached the age of about 30 years, the selection can no longer be as harsh as currently, and our general model allows for success rates of 81% in the tenure-track evaluation. As we have decided to avoid fractional numbers as far as possible, this somewhat overshoots the target success rate of 75% for the general model. Both tenure-track probabilities multiplied result in a success rate of 17% in becoming a professor after having achieved a PhD.

The tenure-track social science department raises these probabilities from the status quo of 11% (Table 7) to 32% (Table 11), the engineering department from 6% (Table 8) to 16% (Table 12) and the natural sciences department from 11% (Table 9) to 16% (Table 13). Again, the importance of these figures lies much less in the final quotas than in a shift toward higher probabilities at later selections, which still take place much earlier than in the status quo.

Much more stringent in this regard is the lecturer model which only includes one selection process after the dissertation, with success rates of 23% (Table 14) compared to the 12% of the general status quo model. The corresponding success rates for the social science department would be 33% (Table 15) instead of 11%, for the engineering department 9% (Table 18, current shares of project funding) or 18% (Table 16) instead of 6%, and for the natural sciences 25% (Table 17) instead of 11%. We thus observe an increase in the overall success rates for all our models. Moreover, due to the variable number of doctoral positions, a good part of these probabilities is not dictated by systematic constraints but rather reflect degrees of freedom for strategic decisions.

## 5 Conclusion

The tenure-track and lecturer models of academic employment presented here show that it is possible to create transparent and fair selection processes for academic staff while at the same time providing enhanced job security. These alternatives do not necessarily increase the chances of an academic career, but they do shift such decisions to a much earlier point in time.

We have been able to demonstrate that, especially in case of the lecturer model, more permanent positions at an earlier stage do not significantly reduce the number of scientific staff that a university can employ. The general tenure-track model results in a reduction of 13.6% of full-time employment equivalents, while reductions within the lecturer model amount to only 3.8%. This is not surprising. Current employment structures do not mean that people are not being employed for longer periods (Kuhnt et al., 2022, p. 53), even if the average duration of a fixed-term contract is only 20 months (Sommer et al., 2022, p. XI). Rather, people work on the basis of consecutive fixed-term contracts, often up to a fairly advanced age, before they finally receive a permanent contract, usually with a professorship, or before they are effectively dismissed at an excessively high rate of about 60%.

The alternatives laid out in our models envisage a significant reduction in the number of doctoral students. This reduction need not lead to a significant decrease in the number of successful doctorate completions. Gassmann (2020, p. 45) has shown that the number of academic staff at universities has more than doubled over the past 25 years while, over the same period, the number of completed dissertations has remained largely stable. Correspondingly, almost two thirds of all academic staff below a professorship do not hold a doctoral degree. These staff members play a crucial role in fulfilling essential university functions, often diverting their focus away from working on their dissertations. By reducing the number of doctoral students in favor of permanent post-doctoral staff, we aim to refocus attention at early stages on the attainment of academic degrees. This shift in balance will ensure that teaching and university organization in particular are carried out by professional staff members who, on the basis of permanent employment contracts, can dedicate their work to advancing research, teaching and thus to the success of universities. Moreover, contrary to the assumption that universities require a permanent throughput of “fresh blood” to ensure innovation, Kyvik and Aksnes (2015) found indicators that publication output mostly grows with age across all analyzed disciplines. In addition, the proposed lecturer models can strongly increase teaching output and therefore greatly improve learning conditions for all students. Longer employment and therefore less turnover furthermore reduce recruitment costs and the loss of organizational knowledge.

Our proposals would benefit from a stronger embedding of professorial decisions in committees encompassing different status groups. We are not questioning the freedom of research but the autonomy of a small leading group to decide alone on the continuation of employment of doctoral students or postdocs. Such a reform could not only counter the abuse of power in academia (Winkler, 2023) and advance professionalized personnel selection processes to avoid discrimination (cf. Steinweg, 2015, p. 16ff). Collective decisions about staff appointment are also helpful in forming units that are large enough to design attractive career paths within single institutions.

We have also included the reduction of project funding rates in our proposals. This is not necessary for the models to work. Rather, it is an additional instrument to increase sustainable employment at universities and reduce transaction costs, as well as counterproductive incentives.

Unfortunately, the statistical data lack information on the age, doctoral status and paid working hours of university staff. Such data are available but are not compiled into the published tables. Better overviews in these respects could significantly improve the understanding of academic career paths. To overcome this lack of information, we had to rely on a number of assumptions and on survey data. However, the models proved to be robust enough so that adjustments would not change the general finding: a sustainable and fair employment structure would benefit both, the universities in general and their employees in particular.

## Data availability statement

The original contributions presented in the study are included in the article/Supplementary material, further inquiries can be directed to the corresponding author.

## Author contributions

MK: Conceptualization, Formal analysis, Methodology, Writing—original draft, Writing—review & editing. PM: Conceptualization, Writing—review & editing. TR: Conceptualization, Writing—review & editing.

## Footnotes

^1^All statistical data stem from the Federal Statistical Office of Germany, using the ICEland portal provided by the German Centre for Higher Education Research and Science Studies (DZHW, https://iceland.dzhw.eu/). The respective data set number (60402) is also always given.

^2^While the Bundestag refrained from a clear definition which qualifications are subject to the WissZeitVG, the federal labor court (Bundesarbeitsgericht, BAG, 7-AZR-573/20 from 02.02.2022) ruled in its decision that basically any qualification fulfills the necessary condition to allow for temporary contracts.

^3^The habilitation is a formal qualification originally necessary to assume a full professorship at a German university.

^4^Based on full time equivalents, Source: Federal Statistical Office of Germany, Fachserie 11 Reihe 4.4.

^5^German Research Foundation (DFG), Personalmittelsätze der DFG für das Jahr 2023, Vordruck 60.12 – 01/23, https://www.dfg.de/formulare/60_12/v/60/12/2023/de.pdf (accessed September 24, 2023).

^6^The higher teaching quotas in the general status-quo model compared to the disciplinary institutes are mainly caused by much higher numbers of permanent employees in the medical sciences.

^7^The lower rate of fixed-term contracts for the general model compared to the disciplinary institutes is caused again by the much higher share of permanent employees in the medical sciences.
