# Supplementary material for: There are alternatives. Models for sustainable employment structures in the German system of higher education
Source: Front Res Metr Anal. 2024 Mar 1;9:1301354. doi: 10.3389/frma.2024.1301354 (PMC10940507; doi:10.3389/frma.2024.1301354)
Supplement: Supplementary file 1 [file Table_1.docx]

Alternative models for sustainable employment structures in the German system of higher education

Supplement

Mathias Kuhnt^1*^, Peter Müßig^2^, Tilman Reitz^3^

^1^Lab for Research in Organization and Differentiation, Faculty of Arts, Humanities and Science, Dresden University of Technology, Dresden, Germany

^2^German Centre for Higher Education Research and Science Studies (DZHW), Research Infrastructure and Methods, Hannover, Germany

^3^Institute of Sociology, Faculty of Arts and Humanities, Friedrich-Schiller-University Jena, Jena, Germany

*** Correspondence:**Mathias Kuhnt
mathias.kuhnt@tu-dresden.de

Table 1: Calculation of employer costs

| **Year of employment** | **Average age** | **Experience level** |  | **Payment employee level E13 brutto 2023** | **Costs employer brutto 2023** | **Status quo averages** | **Lecturer average** | **Lecturer only 10 years average** |
| --- | --- | --- | --- | --- | --- | --- | --- | --- |
| 1 | 27 | 1 |  | 52207 | 62810 |  |  |  |
| 2 | 28 | 2 |  | 56192 | 67606 |  |  |  |
| 3 | 29 | 2 |  | 56192 | 67606 |  |  |  |
| 4 | 30 | 3 |  | 59189 | 71212 |  |  |  |
| 5 | 31 | 3 |  | 59189 | 71212 |  |  |  |
| 6 | 32 | 3 |  | 59189 | 71212 | 68610 |  |  |
| 7 | 33 | 4 |  | 65012 | 77716 |  |  |  |
| 8 | 34 | 4 |  | 65012 | 77716 |  |  |  |
| 9 | 35 | 4 |  | 65012 | 77716 |  |  |  |
| 10 | 36 | 4 |  | 65012 | 77716 |  |  |  |
| 11 | 37 | 5 |  | 73062 | 86619 |  |  |  |
| 12 | 38 | 5 |  | 73062 | 86619 | 80684 |  |  |
| 13 | 39 | 5 |  | 73062 | 86619 |  |  |  |
| 14 | 40 | 5 |  | 73062 | 86619 |  |  |  |
| 15 | 41 | 5 |  | 73062 | 86619 |  |  |  |
| 16 | 42 | 6 |  | 75254 | 89044 |  |  | 83300 |
| 17 | 43 | 6 |  | 75254 | 89044 |  |  |  |
| 18 | 44 | 6 |  | 75254 | 89044 |  |  |  |
| 19 | 45 | 6 |  | 75254 | 89044 |  |  |  |
| 20 | 46 | 6 |  | 75254 | 89044 |  |  |  |
| 21 | 47 | 6 |  | 75254 | 89044 |  |  |  |
| 22 | 48 | 6 |  | 75254 | 89044 |  |  |  |
| 23 | 49 | 6 |  | 75254 | 89044 |  |  |  |
| 24 | 50 | 6 |  | 75254 | 89044 |  |  |  |
| 25 | 51 | 6 |  | 75254 | 89044 |  |  |  |
| 26 | 52 | 6 |  | 75254 | 89044 |  |  |  |
| 27 | 53 | 6 |  | 75254 | 89044 |  |  |  |
| 28 | 54 | 6 |  | 75254 | 89044 |  |  |  |
| 29 | 55 | 6 |  | 75254 | 89044 |  |  |  |
| 30 | 56 | 6 |  | 75254 | 89044 |  |  |  |
| 31 | 57 | 6 |  | 75254 | 89044 |  |  |  |
| 32 | 58 | 6 |  | 75254 | 89044 |  |  |  |
| 33 | 59 | 6 |  | 75254 | 89044 |  |  |  |
| 34 | 60 | 6 |  | 75254 | 89044 |  |  |  |
| 35 | 61 | 6 |  | 75254 | 89044 |  |  |  |
| 36 | 62 | 6 |  | 75254 | 89044 |  |  |  |
| 37 | 63 | 6 |  | 75254 | 89044 |  |  |  |
| 38 | 64 | 6 |  | 75254 | 89044 |  |  |  |
| 39 | 65 | 6 |  | 75254 | 89044 |  |  |  |
| 40 | 66 | 6 |  | 75254 | 89044 | 88784 | 87355 |  |
